# Customized Pulmonary Artery Stent for Management of Complex Coronary Artery Fistula

**DOI:** 10.1016/j.jaccas.2025.104748

**Published:** 2025-08-27

**Authors:** Rawan Amir, John Thomson, Ari Cedars, Stacy Fisher

**Affiliations:** a Division of Cardiology, Department of Medicine, Johns Hopkins Hospital School of Medicine, Baltimore, Maryland, USA; b Division of Pediatric Cardiology, Department of Pediatrics, Johns Hopkins School of Medicine, Baltimore, Maryland, USA

**Keywords:** congenital heart defect, coronary vessel anomaly, percutaneous coronary intervention, stents

## Abstract

**Background:**

Coronary artery fistulas (CAFs) are rare anomalies that can cause ischemia, arrhythmias, and sudden cardiac death. Conventional closure techniques often fail in anatomically complex cases.

**Summary:**

We present the first-in-human use of a customized, partially covered G-Armor pulmonary artery (PA) stent to manage a left main–to–right pulmonary artery fistula in a patient with Shone complex. The intervention was planned using a three-dimensionally printed model, allowing precise stent customization to avoid obstruction of key pulmonary branches.

**Discussion:**

This case shows the feasibility of using customized endovascular devices to address complex CAFs in high-risk patients. The intervention improved antegrade coronary perfusion and clinical outcomes.

**Novelty:**

To the best of our knowledge, this is the first reported case of a customized partially covered PA stent for CAF management.

**Take-Home Message:**

In anatomically complex and high-risk CAFs, customized stenting offers a viable solution when conventional therapies fail.

## Visual Summary

**Figure:**
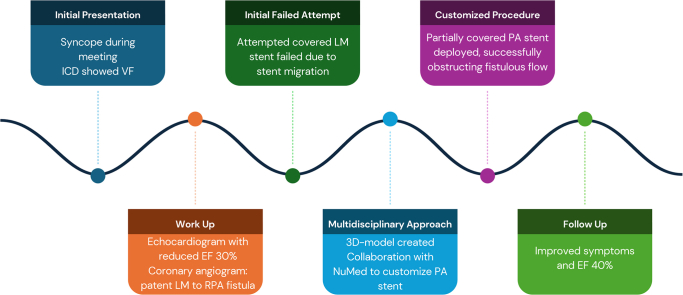

**Visual Summary:**
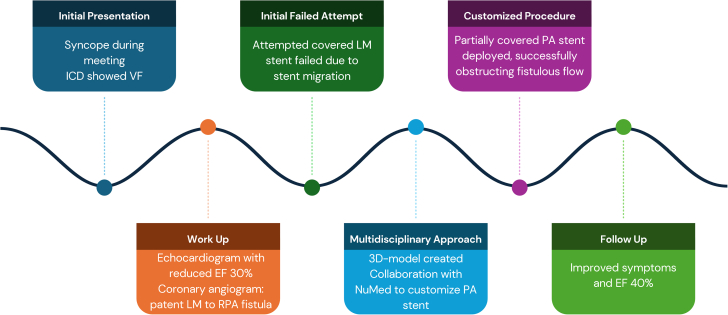
Timeline of Key Events From Initial Presentation to Follow-Up After Intervention 3D = three-dimensional; EF = ejection fraction; ICD = implantable cardioverter-defibrillator; LM = left main; PA = pulmonary artery; RPA = right pulmonary artery; VF = ventricular fibrillation.

Coronary artery fistulas (CAFs) are rare vascular anomalies that can pose unique therapeutic challenges. Herein, we report a novel solution to a complex left main (LM)–to–right pulmonary artery (RPA) fistula in a patient with Shone complex. The CAF was managed with a customized, partially covered 8.4-cm G-Armor pulmonary artery stent after failure of standard approaches.

**Take-Home Messages**

• Customized, partially covered stents can offer a safe and effective solution for complex coronary artery fistulae when standard interventions fail.
• Patient-specific device design may reduce the need for high-risk surgical interventions.
• Multidisciplinary planning and 3D modeling are essential tools in tailoring interventions to complex congenital anatomy.

## Case Presentation

A 63-year-old man presented after a witnessed syncopal episode during a virtual meeting. The patient has a complex cardiovascular history, including Shone complex with a bicuspid aortic valve, stenotic parachute mitral valve, aortic coarctation, subaortic stenosis surgically resected during childhood, and a LM-to-RPA fistula. This latter diagnosis had resulted in ischemic cardiomyopathy, with an ejection fraction (EF) of 30% and ventricular fibrillation status post–intracardiac defibrillator placement with subsequent biventricular pacemaker upgrade. As a result of these sequelae, the fistula had been previously treated with coiling approached from the coronary artery.

On presentation, he denied any prodrome to his syncopal event and reported regaining consciousness within a few minutes. Review of systems was significant for decline in exercise capacity and occasional anginal-quality chest pain. On arrival, vital signs, physical examination, and laboratory tests including cardiac biomarkers were unremarkable. Transthoracic echocardiogram showed normal left ventricular size, reduced function with an EF of 30%, severe hypokinesis of mid to distal anterior and anterolateral wall, apical akinesis with thinning, and mild to moderate mitral stenosis, with mean diastolic gradient of 7 mm Hg at a heart rate of 66 beats/min. Device interrogation revealed an episode of ventricular fibrillation at 260 beats/min, appropriately treated with a 36-J shock, coinciding with his syncopal event.

**Figure 1:**
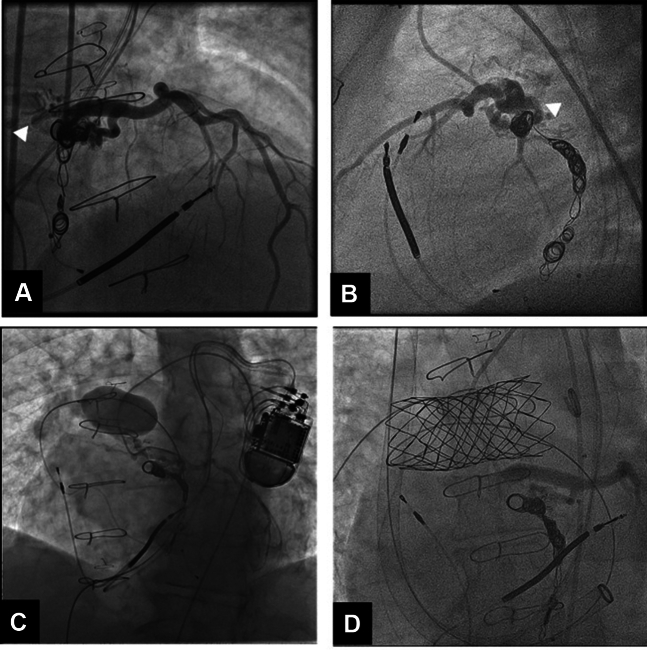
Coronary Angiogram for CF Closure Coronary angiograms showing (A and B) LM-to-RPA fistula (arrowhead), (C) occlusion of PA with balloon obstructing fistulous flow from the LM into the PA, and (D) no evidence of fistulous flow after PA stent deployment while injecting the left coronary system. LM = left main; PA = pulmonary artery; RPA = right pulmonary artery.

Cardiac catheterization was performed to investigate a possible ischemic etiology for the ventricular fibrillation and revealed minimal nonobstructive coronary artery disease. It demonstrated persistent fistulous flow from the LM to the RPA via existing coiled channels and new collateral branches, creating a steal phenomenon compromising antegrade flow into left anterior descending artery (Figures 1A and 1B). An attempt to cover the fistula origin using a 4.8 × 16 mm covered GraftMaster LM stent (Abbott) was unsuccessful because of stent migration into the distal LM, possibly due to a dilated proximal LM measuring 7.0 mm.

**Figure 2:**
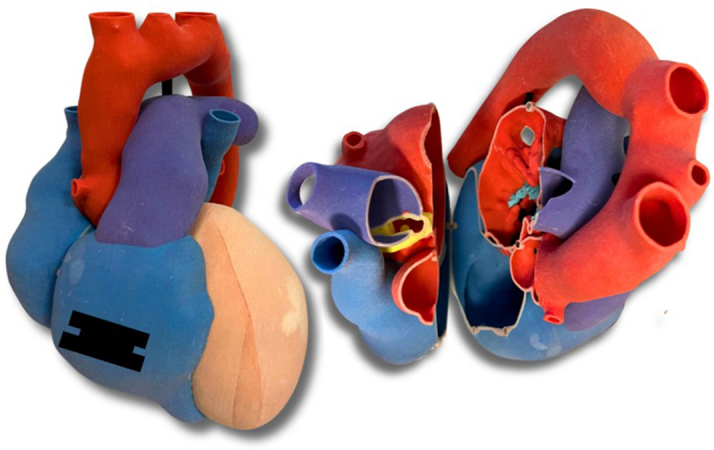
Cardiac 3D Model (Left) Anterior view and (Right) superior view opened along the long axis revealing the LM-to-RPA coronary artery fistula. Red indicates the aorta and left atrium, pink indicates the left ventricle, blue indicates the right atrium and right ventricle, purple indicates the pulmonary artery, and yellow indicates the coronary artery fistula. 3D = three-dimensional; LM = left main; RPA = right pulmonary artery.

**Figure 3:**
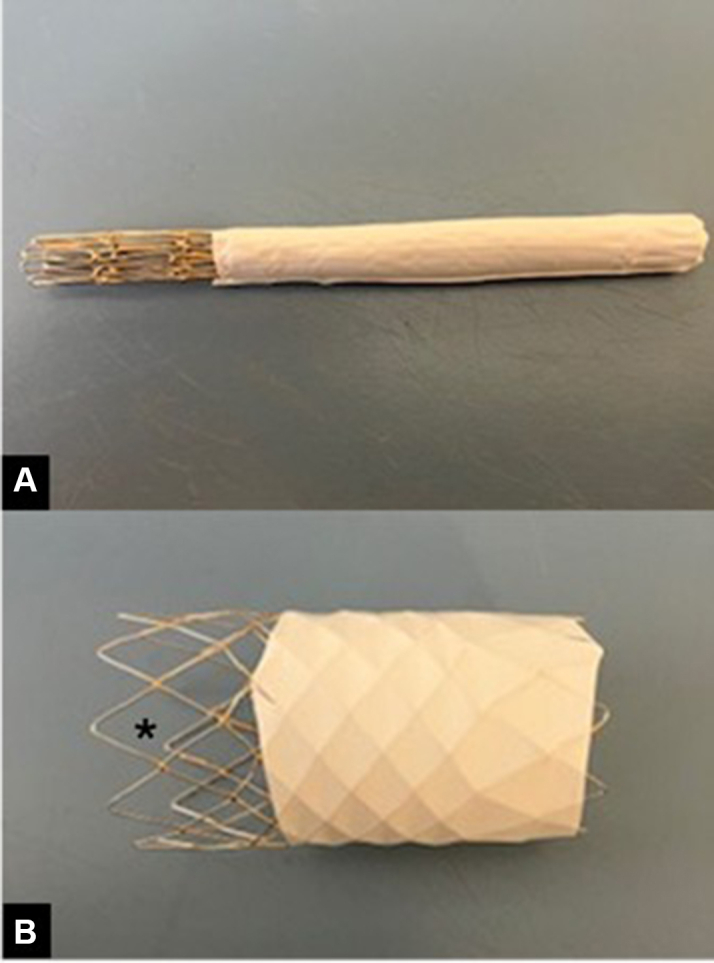
Customized, Partially Covered 8.4-cm G-Armor Stent With Uncovered Distal Zig Stent crimped (A) predeployment and (B) postdeployment demonstrating expansion of covered portion while maintaining an uncovered distal zig (∗) to optimize hemodynamics.

Given the patient's high surgical risk with a history of multiple sternotomies, as well as his preference to avoid redo sternotomy, a multidisciplinary team consisting of adult congenital cardiologists, interventional cardiologists, and cardiac surgeons opted to take the approach of obstructing the fistula termination site at the RPA. Computed tomography of the chest, however, demonstrated large branches from the RPA near the fistula termination site, such that covered stent placement could jail and compromise flow to a significant portion of the right lung. A three-dimensional (3D) model was printed to assist with planning (Figure 2), and in collaboration with NuMed, we created a customized, partially covered 8.4-cm G-Armor stent (Figure 3). Food and Drug Administration exception and compassionate Institutional Review Board approval were obtained for single-case use.

During the intervention, an angiogram of the pulmonary artery (PA) confirmed the presence of a large right upper lobe branch close to the fistula termination site. A 34-mm balloon-in-balloon device, selected given its enhanced positional stability during inflation, was advanced and test-inflated to 2 atm in the RPA. Simultaneously, a Judkins left catheter was positioned in the LM and injected, confirming obstruction of shunt flow, with return of fistulous flow with balloon deflation (Figure 1C). The G-Armor stent was then hand mounted on the same balloon, with the distal zig left uncovered to avoid obstruction of the RPA branch. A 26-F 65-cm DrySeal sheath (Gore Medical) was advanced via the right femoral vein, however it was too short and terminated in the main PA. Alternate access via right internal jugular vein was obtained, and the RPA was accessed using the 26-F 65-cm DrySeal sheath. A second G-Armor stent was mounted on a second 34-mm balloon-in-balloon device and advanced to the target location in the RPA. Numerous checks to ensure correct positioning were performed using a 6-F pigtail catheter from the contralateral femoral vein, to avoid jailing the left PA. The balloon was then serially inflated until the stent was completely deployed. Final LM angiogram showed complete obstruction of fistulous flow, with improved antegrade flow into the left anterior descending artery (Figure 1D). At 2-month follow-up, the patient reported no recurrent syncope and improved exercise capacity. Subsequent transthoracic echocardiogram showed improvement in left ventricular function, with an EF of 40%.

## Novelty of This Approach

CAFs are rare vascular connections between the coronary arteries and cardiac chambers or systemic/pulmonary vessels. While most CAFs are hemodynamically insignificant and patients are asymptomatic, others may result in myocardial ischemia, arrhythmias, ventricular dysfunction, endarteritis, or cardiac chamber enlargement.^1^

Large CAFs, regardless of symptoms, along with small to moderate CAFs in the presence of myocardial ischemia, ventricular dysfunction, arrhythmias, or endarteritis, should be closed using transcatheter or surgical approaches.^2^ Current transcutaneous options, including coils, vascular plugs, and covered stents, are considered first-line therapies in patients with suitable anatomy. Despite success rates nearing 90% with these strategies, they are not without complications, with rates of device embolization, postprocedural myocardial ischemia, arrhythmia, and hemorrhagic side effects given need for postprocedure anticoagulation or antiplatelet therapy ranging from 18% to 34%.^3^^,^^4^ Additionally, initial failure of these strategies is seen particularly with more complex lesions; and even after initial success, recanalization has been reported in about 15% of patients.^3^

To the best of our knowledge, this is the first case reporting use of a customized partially covered PA stent for management of a complex CAF after failure of traditional therapies. Our approach highlights the importance of individualized care and multidisciplinary consideration. In addition, use of a 3D model was critical to understanding the anatomy and spatial relationship between the CAF and the RPA, which allowed precise tailoring of the stent, planning of the procedure, and anticipation of potential challenges.

## Future Directions

This case shows that personalized endovascular devices may broaden therapeutic options for complex CAFs, especially in patients with congenital heart disease with prior sternotomies or challenging vascular anatomy. Further exploration of tailored device development could benefit patients for whom standard therapies are ineffective or contraindicated.

## Conclusions

This case highlights the complexity of managing CAF and the importance of considering innovative approaches when standard therapies fail. It also highlights the value of integrating advanced 3D technology in planning complex procedures and of clinician and industry development.

## Funding Support and Author Disclosures

The authors have reported that they have no relationships relevant to the contents of this paper to disclose.
